# Extraction and characterization of starch from low-grade potatoes and formulation of gluten-free cookies containing modified potato starch

**DOI:** 10.1016/j.heliyon.2023.e19581

**Published:** 2023-09-01

**Authors:** Syed Mueez Ali, Yumna Siddique, Samina Mehnaz, Muhammad Bilal Sadiq

**Affiliations:** Kauser Abdulla Malik School of Life Sciences, Forman Christian College (A Chartered University), Lahore, 54600, Pakistan

**Keywords:** Potato starch, Modified starch, Water steeping, Gluten-free cookies

## Abstract

Potatoes are among the leading staple crops due to nutritional value and high demand. The undersized and damaged potatoes are considered low grade and mainly dumped as a waste or used in animal feed. The study aimed to extract starch from low grade potatoes, its modification to improve the starch properties and formulation of gluten free cookies using modified starch (MS). The starch was extracted from low-grade potatoes of three varieties known as Asterix, Kruda and Mosaic, using the water steeping method. The native starch (NS) was modified using lintnerization and repetitive autoclaving. MS contains high amylose content which is associated with health benefits. NS and MS were characterized for amylose content, color attributes, granular morphology, water solubility index (WSI), water absorption index (WAI), thermogravimetric analysis (TGA) and Fourier transform infrared spectrometer (FTIR) analysis. Gluten-free cookies were formulated by adding potato NS and MS. The cookies were characterized by sensory evaluation, proximate and textural analysis. The starch yield extracted from three different varieties of potatoes i.e. Asterix, Kruda, Mosaic was 11.53%, 11.32% and 11.24%, respectively. The amylose content of potato starch was significantly (p < 0.05) increased for all varieties (33.61–37.74%) after modification of NS, which was in the range of 25.71–26.60% for different potato varieties. The granules of MS were observed as amorphous structures in comparison to NS granules with smooth surfaces. The addition of MS significantly (p < 0.05) decreased the hardness of the cookies in comparison to NS. Overall, no significant difference was observed in the sensory attributes of control, NS and MS containing cookies. Therefore, in comparison to other dietary fibers, MS can be used as a functional ingredient in food products without compromising the texture and sensory attributes.

## 1 Introduction

The world's most grown staple crops encompass the potato (*Solanum tuberosum*), which belongs to the Solanaceae family. In terms of global production, the potato crop ranked third after wheat and rice. Food and Agriculture Organization (FAO) of the United Nation reported global production of potatoes over 370 million metric tons [1]. Potato achieves this major position among staple crops because of its highly nutritious properties, potential industrial and domestic usage both in processed and non-processed forms, and easy accessibility to consumers from low-income backgrounds [2]. The nutritional benefits of potatoes impart a vital role in fulfilling energy requirements at low cost in under-developed countries [3]. The potato tuber comprises of several essential amino acids, most importantly lysine, high dietary fiber and starch content, and low-fat content [4]. The high starch content makes potatoes a basic raw material for several food industries to make value-added products like chips, fries, flakes and pre-peeled potatoes [5].

Pakistan has good climatic conditions for potato crop growth and annual production of 5.87 million tons was reported in 2021 [6]. Starch is the major component (12–15%, dry basis) of potatoes after the water and serves as an indicator of crop quality [7]. In comparison to different starch sources, potato starch has unique physicochemical properties, high purity, low fat and protein content, long chains of amylose and amylopectin. The presence of phosphate ester group and low-fat moiety in potato starch improves its gel forming ability [7].

Approximately 30% of the potato production is discarded during harvesting and storage due to undersized and irregular shape [8]. The discarded potatoes are primarily used for animal feed [9]. However, low-grade or discarded potatoes can be used as a source of starch as the carbohydrate content remains intact in the absence of microbial infection [8]. The physicochemical and functional properties of starch can be improved by increasing amylose content. It can be increased by treating the native starch with physical, chemical or enzymatic methods to make it resistant starch (RS) or modified starch. RS is sum of starch and its degraded products which are not absorbed in intestine and serve as a substrate for probiotics in large intestine [10]. Gluten is a major wheat protein which is associated with celiac disease and gluten allergy [11]. Gluten free products are in high demand due to increased consumer awareness. Cereal based gluten-free cookies and breads are consumed most as a replacement to wheat gluten [12]. Corn, rice and potato flour are frequently used in the formulation of gluten-free products [13].

RS has various health benefits due to resistance towards enzymatic and temperature treatments. The high amylose content of RS improves its physicochemical properties such as gel strength, swelling capacity, viscosity, and film-forming ability [14]. This aimed to extract the starch from discarded potatoes and conversion of NS into MS. MS was characterized by its functional and physicochemical characteristics. Furthermore, MS was used for the formulation of gluten free functional cookies, which were evaluated for the organoleptic characteristics.

## 2 Materials and methods

### 2.1 Sample collection

Undersized low-grade potatoes (10 kg each) of three different varieties (Asterix, Kruda, Mosaic) harvested in Punjab province of Pakistan were collected from the local distributors. The samples were washed with tap water to remove surface dirt, and sprouts were removed using knife. Potatoes were stored in cold storage (5 ± 1 °C) and all samples were utilized within one week of storage.

### 2.2 Starch extraction

The starch was extracted from low-grade potatoes using water steeping method [8]. The potatoes were manually peeled and cut into small pieces (400 g), followed by blending with water (1:2 w/v) in a blender (Philips, Cucina Series, Shanghai, China). The slurry was agitated for 90 min at 200 rpm, followed by sieving consecutively through 150 μm and 80 μm pore size sieves. The residual flesh on the sieves was further washed with water to drag the remaining starch. The filtrate was left for 24 h at 4 °C to allow the starch sedimentation. The starch was separated by decantation and washed twice with water. Finally, the NS was dried in a hot air oven at 40 °C for 48 h. The dried starch was stored in zip-lock bags for further analysis.

### 2.3 Proximate analysis of starch

The proximate composition of starch was estimated for moisture (AOAC 934.01), ash (AOAC 942.05), protein (AOAC 981.10), and crude fat (AOAC 920.39) contents by using standard AOAC methods [15]. Carbohydrate content was determined by subtracting the sum of all components from 100.

### 2.4 Modification of starch

The potato starch was modified using a combination of lintnerization (acid hydrolysis) and heat treatment as described by Shrestha et al. [10], with slight modifications. The potato starch was suspended in 2 N HCl (1:1.5, w/v) and incubated at 40 °C in a shaking incubator for 3 h. After the incubation pH was adjusted to 6.5 using 10% (w/v) NaOH solution, followed by drying at 40 °C for 24 h. The lintnerized starch was suspended in water (1:10, w/v), gelatinized at 85 °C for 30 min, then autoclaved at 135 °C for 30 min and refrigerated at 4 °C for 24 h. The autoclaving and cooling cycle was repeated three times to obtain the modified starch and then dried in a hot air oven at 50 °C and grounded by a grinder.

### 2.5 Characterization of modified and native potato starch

NS and MS were characterized for color attributes, granular morphology, water solubility index (WSI), water absorption index (WAI), thermogravimetric analysis (TGA), and FTIR analysis.

### 2.6 Color analysis

The different starches were subjected to color analysis using hand-held colorimeter (CR-10, Minolta, Osaka, Japan), and the results were expressed in the CIE Lab system (L*, a*, b*). All analyses were conducted in 5 replicates at room temperature under the same light conditions.

### 2.7 Granular morphology

The granule morphology of NS and MS was analyzed under the light microscope. The starch sample (20 mg) was suspended in 2 ml distilled water and a drop of the suspension was placed on a microscopic slide. After adding a drop of iodine solution, the slide was observed under the light microscope (Meiji Techno, Japan) at a magnification of 400× [16].

### 2.8 FTIR analysis of starch

Chemical fingerprinting of each starch sample was analyzed by FTIR spectrometer (Bruker, ALPHA II, Germany) in the range of 650 cm^−1^ to 4000 cm^−1^ wavenumbers.

### 2.9 WAI and WSI

WSI and WAI of the starches were determined by following Gerçekaslan [17] with slight modification. One gram (W1) of starch was transferred in a pre-weighed 50 ml falcon tube (W2) and 30 ml of distilled water was added to form a suspension. The suspension was incubated in shaking water bath at 200 rpm for 30 min at 30 °C and then centrifuged at 1500×*g* for 10 min. The supernatant was transferred to pre-weighed petri plates (W3) and the falcon tube with pallet was weighed (W4). The petri plates were placed in a hot air oven at 105 °C for 24 h and the final weight of the petri plates (W5) was recorded. Equations (1), (2)) were used to calculate WAI and WSI, respectively.

(1) $$\text{WAI}\;\left(g/g\right)=\frac{W4-W2}{W1}$$

(2) $$\text{WSI}\;\left(\%\right)=\frac{W5-W3}{W1}\times 100$$

### 2.10 Thermogravimetric analysis

Thermogravimetric analysis (TGA) of the starch was carried out by using thermogravimetric analyzer (SDT Q600, Ta Instruments, USA). For TGA analysis starch suspension (6–7 mg) was heated from 30 °C to 600 °C at the rate of 20 °C/min. The total weight loss was recorded with the increase in temperature.

### 2.11 Amylose content

Thee iodine calorimetric method was used to determine the amylose content of native and modified starches [18]. Starch (100 mg) was taken in volumetric flask and added with 1 ml of 95% ethanol and 9 ml of 1 N NaOH. The mixture was heated at 100 °C for 10 min, and the final volume was adjusted to 100 ml after cooling the reaction mixture to room temperature. An aliquot of 2.5 ml was taken from reaction mixture in another volumetric flask and mixed with 1 ml of 1 N acetic acid and 1 ml of iodine solution. The final volume was raised to 50 ml using distilled water and after shaking sample was incubated for 20 min in dark. The absorbance was read at 620 nm using UV–Visible spectrophotometer (UV-1900i, Shimadzu, Japan). The amylose content of the starch samples was determined using the amylose standard curve prepared at different concentrations (0–40%).

### 2.12 Formulation of gluten-free cookies incorporated with modified potato starch

The gluten-free cookies were prepared by following the protocol of Mohammadi et al. [19] with some modifications. The gluten-free cookies were prepared using the ingredients; gram flour (55 g), butter (35 g), starch/modified starch (15 g), sugar (20 g), water (30 ml), egg (5 g) and baking soda (0.5 g). The control cookies were prepared by adding additional gram flour in place of MS. The ingredients were mixed to form a dough which was rested at 4 °C for 30 min. The dough was cut using a mold and baked at 175 °C for 20 min in a baking oven. The cookies were cooled to room temperature and stored in ziplock bags for further analysis.

### 2.13 Texture analysis

The texture analysis of cookies was determined by using texture analyzer (TX-700, LAMY Rheology Instruments, France). The texture profile of the cookies was evaluated by using a cylindrical probe at a speed of 2 mm/s and compression distance was set at 3 mm. The triggering force was 50 g using a load cell of 250 N [20].

### 2.14 Proximate analysis of cookies

The proximate composition (moisture, ash, crude fat, crude protein and total carbohydrate content) of gluten-free cookies was determined by using AOAC standard methods [15].

### 2.15 Sensory analysis

Sensory analysis of freshly baked gluten free cookies was conducted by 9 point hedonic scale (1 = dislike extremely, to 9 = like extremely) with 25 panelist with food science background. Samples were coded with 3-digit numbers and panelists were asked to evaluate each sensory parameter (texture, odor, sweetness, crispiness, color, appearance, taste and overall acceptability) [21]. Informed consent was obtained from all the participants.

### 2.16 Statistical analysis

One-way analysis of variance (ANOVA) and Tukey's HSD tests were used to find the significant differences among mean observations (p < 0.05) using SPSS statistical software package (SPSS, version 23.0, USA).

## 3 Results and discussion

### 3.1 Starch yield and proximate composition

The starch yield extracted from three different varieties of potatoes, i.e. Asterix, Kruda and Mosaic, was 11.53%, 11.32% and 11.24%, respectively. Nawaz et al. [7] extracted starch from peeled potatoes using the water steeping method and, starch yield was found in the range of 12–12.7% for different varieties. In this study, relatively lower starch yield can be attributed to the use of multiple layering of the sieves and washing of starch to ensure the removal of impurities.

Proximate analysis of potato starches revealed that the moisture content, ash, crude fat, protein and carbohydrate content were in the range of 12.66–14.55%, 0.14–0.30%, 0.29–1.65%, 1.03–1.16% and 82.27–85.87%, respectively (Table 1). The carbohydrate content of Mosaic (85.87%) and Kruda potato starch (85.23%) was significantly higher than the Asterix (82.27%). Asterix starch contained significantly high moisture content in comparison to Mosaic and Kruda potato starch, which might be due to different potato variety or maturity stage. Martínez et al. [22] determined the proximate composition of several native potato varieties grown in Peru and reported the range of moisture content (10.20–15.79%), protein content (0.10–0.44%), crude fat (0–0.21%) and ash content (0.16–0.45%).

**Table 1:** Potato starch yield and proximate composition.

Components	Asterix	Kruda	Mosaic
Yield (%)	11.53 ± 0.18^a^	11.32 ± 0.34^a^	11.24 ± 0.50^a^
Moisture (%)	14.55 ± 0.35^a^	12.67 ± 0.09^b^	12.66 ± 0.07^b^
Ash (%)	0.30 ± 0.02^a^	0.27 ± 0.02^a^	0.14 ± 0.02^b^
Fat (%)	1.65 ± 0.35^a^	0.74 ± 0.11^b^	0.29 ± 0.01^c^
Protein (%)	1.16 ± 0.11^a^	1.03 ± 0.06^a^	1.06 ± 0.12^a^
Carbohydrate (%)	82.27 ± 0.63^b^	85.23 ± 0.15^a^	85.87 ± 0.23^a^

Different small superscripts (a - c) within a row indicate mean values which are significantly different (p < 0.05).

### 3.2 Characterization of modified and native potato starch

#### 3.2.1 Color analysis

Color attributes (CIE, L*, a* b* system) of the native and modified starches are summarized in Table 2. Lightness (L* value) of the starch decreased after the conversion of NS into MS. Whereas, redness (a* value) and yellowness (b* value) of starches significantly increased after the starch modification. Ali et al. [23] reported a decrease in L* value of potato starch from 95.83 to 79.44 after the extrusion treatment at 130 °C. High temperature treatment promotes the non-enzymatic browning of starch which contributes to a decrease in lightness (L* value) [24].

**Table 2:** Color attributes, amylose content (%) water solubility index (WSI) and water absorption index (WAI) of native and modified potato starches.

Potato Starch Variety	L*	a*	b*	WSI (%)	WAI (g/g)	Amylose content (%)
Native Asterix	89.60 ± 0.14^c^	1.34 ± 0.09^b^	8.28 ± 0.08^c^	33.79 ± 3.85^c^	9.88 ± 1.21^b^	25.74 ± 2.09^b^
Modified Asterix	80.76 ± 0.91^d^	4.68 ± 0.19^a^	18.98 ± 0.43^a^	68.31 ± 3.55^a^	3.03 ± 0.03^c^	33.61 ± 0.22^a^
Native Kruda	94.90 ± 0.19^a^	0.92 ± 0.04^c^	6.70 ± 0.12^d^	35.89 ± 3.89^c^	14.54 ± 0.84^a^	26.60 ± 2.36^b^
Modified Kruda	80.60 ± 0.40^d^	4.42 ± 0.16^a^	16.70 ± 0.32^b^	67.74 ± 2.59^a^	3.97 ± 0.04^c^	37.74 ± 0.15^a^
Native Mosaic	91.94 ± 0.09^b^	1.16 ± 0.09b^c^	7.18 ± 0.08^d^	49.33 ± 4.57^b^	9.60 ± 1.04^b^	25.71 ± 1.99^b^
Modified Mosaic	78.0 ± 1.18^e^	4.420 ± 0.26^a^	17.20 ± 0.55^b^	59.16 ± 0.41^a^	4.58 ± 0.22^c^	37.64 ± 0.43^a^

Different small superscripts (a–e) within a column indicate mean values which are significantly different (p < 0.05).

#### 3.2.2 Granular morphology

**Fig. 1:**
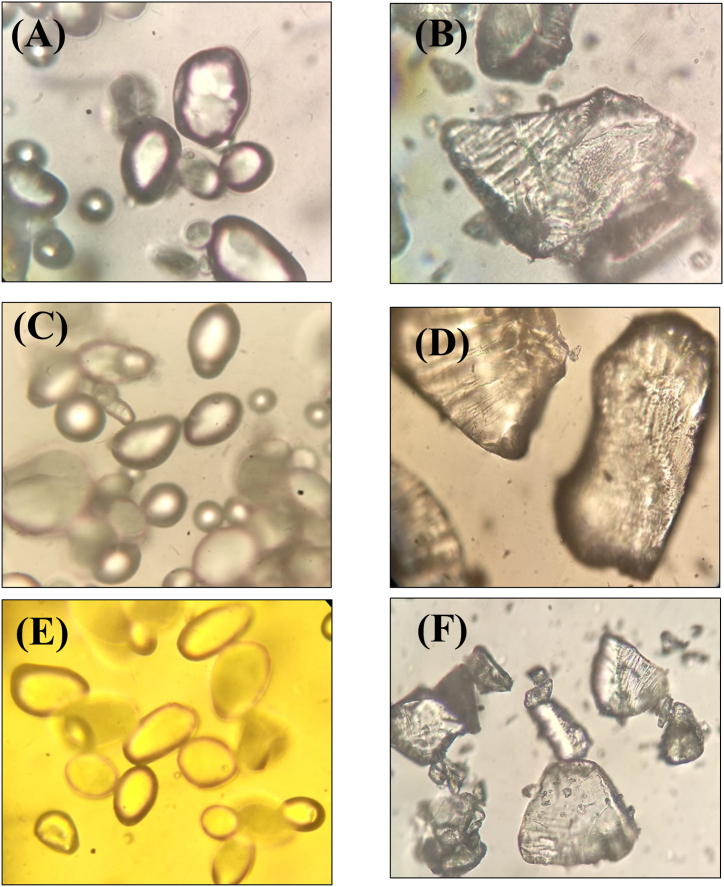
Granular morphology of native and modified potato starches observed by light microscopic. A = Native Asterix starch, B = Modified Asterix starch, C = Native Kruda starch, D = Modified Kruda starch, E = Native Mosaic starch, F = Modified Mosaic starch.

The granular morphology of native potato starch was significantly changed after the modification. Native potato starches exhibit oval to rod shaped granules with uniform surface [Fig. 1(A, C and E)]. However, after the starch modification, granular morphology became irregular and starch granules were observed as amorphous structures due to the retrogradation [Fig. 1(B, D and F)] [10]. Prolonged treatment of potato starch at high temperature resulted in loss of starch granule integrity, which was associated with granule swelling and amylose release. The high degree of starch granule swelling was reported to be linked with high water distribution and weakening of interactions among starch molecules, which ultimately resulted in starch granule destruction [25]. Future investigation of granular morphology by scanning electron microscope can provide a comprehensive understanding of surface changes in starch granules.

#### 3.2.3 WAI and WSI

WAI corresponds to the water holding capacity of starch and its stability in aqueous dispersion. WAI of modified starches was significantly lower than the native potato starches. The reduction in WAI of MS was attributed to the degradation of the polymer during the modification process which reduced the aqueous absorption [23].

WSI is a measure of starch degradation, conversion and soluble component of starch released as a result of modification or processing conditions [26]. It was observed that WSI of modified potato starches was significantly higher than the native starches (Table 2).

#### 3.2.4 Amylose content

The amylose content of native potato starches was in the range of 25.71–26.60% and was not significantly different for Asterix, Kruda and Mosaic starch. After the modification there was a significant increase in the amylose content of all potato starches, which indicated the conversion of amylopectin chains into amylose. Amylose content of modified Asterix, Kruda and Mosaic starches was 33.61, 37.74 and 37.64%, respectively (Table 2). Enzymatic treatment, acid hydrolysis and heat treatments were reported to increase the amylose content of the starch significantly. The enzymatic hydrolysis of retrograded gelatinized native banana starch resulted in an increase in amylose content from 23.10 to 40.46% [27], however, the combination of lintnerization and heat treatment was higher in the amylose content from 30.82 to 55.79% [10].

#### 3.2.5 FTIR analysis

**Fig. 2:**
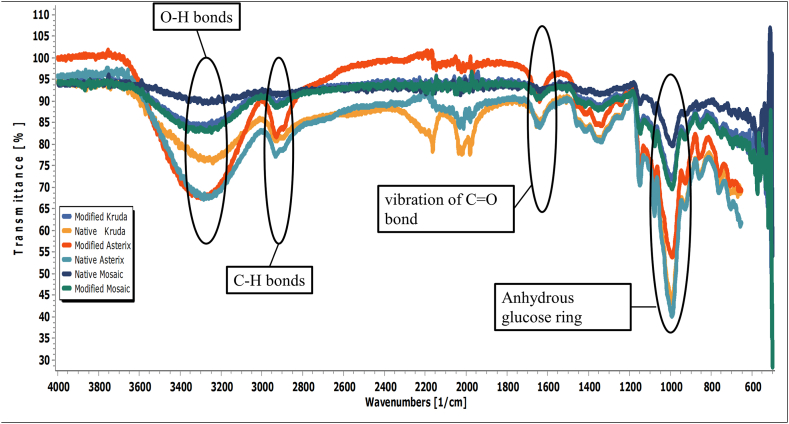
FTIR analysis of native and modified potato starches.

FTIR spectra of native starches changed after the modification, which indicated structural variation among native and modified starches (Fig. 2). Peaks between 980 and 1040 cm^−1^ correspond to anhydrous glucose ring and it was significantly different after the modification of native potato starches. Another significant peak (1636–1656 cm^−1^) indicating the water absorbed in amorphous region (stretching vibration of C]O bond) was changed after the structural modification of starch [28]. The peaks in the range of 3280–3350 cm^−1^ and 2929–2935 cm^−1^ correspond to O–H and C–H bonds, respectively. The intensity of these peaks was different for native and modified starches. The variations in FTIR spectra of native and modified starches confirmed the structural variations in starch as a result of modification treatment [10].

#### 3.2.6 TGA analysis

**Fig. 3:**
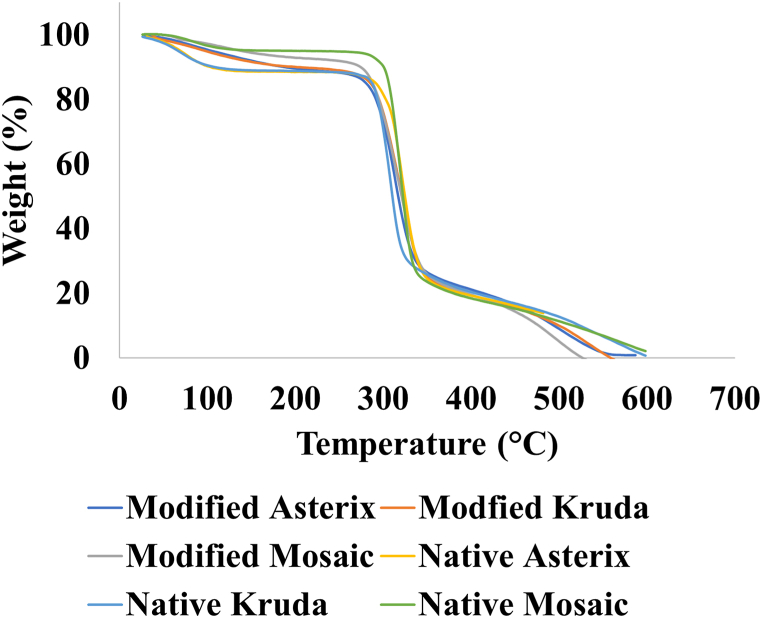
Thermogravimetric analysis of native and modified potato starches.

The thermal decomposition of native and modified potato starches was determined by TGA (Fig. 3). Initially, there was slight weight loss till 270 °C which was associated with evaporation of moisture [29]. The initial weight loss of modified Asterix, Kruda and Mosaic starch was 12.95%, 12.19% and 9.26%, respectively, which was high in comparison to native starches, 12.2%, 12.14% and 5.42%, respectively. A rapid second weight loss was observed from 270 to 345 °C, which was associated with starch pyrolysis. Weight loss above 355 °C was associated with the decomposition of lignin and cellulose derivatives present in starch [30]. At 480 °C, weight loss of modified Asterix, Kruda and Mosaic starch was higher than the native potato starches. The thermal decomposition of starch is mainly influenced by amylose and amylopectin contents. An increase in the amylose to amylopectin ratio of the starch can decrease the thermal decomposition temperature as amylopectin chains require more energy to breakdown due to high molecular weight. The starch with more amylose content was reported to have low thermal stability [31].

#### 3.2.7 Gluten-free cookies

The proximate composition of gluten-free cookies fortified with modified starches is summarized in Table 3. There was no significant difference in the fat and ash content of control and potato starch added cookies. The protein content of the control cookies was significantly higher than the other cookies, which was due to the reason that the NS or MS starch was incorporated as a replacement of gram flour which reduced the overall protein content of gluten free cookies. Similarly, the carbohydrate content of the NS or MS starch incorporated cookies was significantly higher than the control cookies.

**Table 3:** Proximate composition of gluten free cookies incorporated with potato starch.

Gluten free cookies	Moisture (%)	Ash **(**%**)**	Fat **(**%**)**	Protein **(**%**)**	**C**arbohydrate **(%)**
Control	7.07 ± 0.31^a^	1.42 ± 0.26^a^	18.43 ± 2.31^a^	7.11 ± 0.35^a^	65.97 ± 1.81^b^
Native Kruda	6.28 ± 0.68^ab^	1.21 ± 0.31^a^	16.30 ± 0.96^a^	5.73 ± 0.50^b^	70.47 ± 1.60^a^
Native Mosaic	4.79 ± 0.42^d^	1.16 ± 0.34^a^	19.25 ± 0.74^a^	5.47 ± 0.40^bc^	69.33 ± 1.80^bc^
Native Asterix	5.47 ± 0.10^bcd^	1.34 ± 0.20^a^	17.10 ± 1.33^a^	4.90 ± 0.70^bc^	71.19 ± 2.12^a^
Modified Kruda	5.93 ± 0.45^bc^	1.18 ± 0.02^a^	19.63 ± 1.88^a^	4.25 ± 0.49^c^	69.00 ± 1.44^bc^
Modified Mosaic	5.11 ± 0.20^cd^	1.36 ± 0.10^a^	19.50 ± 1.15^a^	5.24 ± 0.34^bc^	68.79 ± 1.35^bc^
Modified Asterix	5.79 ± 0.3^bcd^	1.04 ± 0.35^a^	16.70 ± 0.82^a^	4.71 ± 0.33^bc^	71.76 ± 0.78^a^

Different small superscripts (a–d) within a column indicate mean values which are significantly different (p < 0.05).

#### 3.2.8 Texture analysis

The addition of MS significantly (p < 0.05) decreased the hardness of the cookies in comparison to native starch (Table 4). Cookies with native starch of Asterix, Kruda and Mosaic showed hardness values of 218.73 N, 209.83 N and 180.16 N, respectively, which were significantly reduced to 156.69 N, 192.57 N and 162.78 N, respectively. Similarly, the gumminess of cookies, containing MS was lower than the cookies containing native starch. The cohesiveness and gumminess of native Asterix starch containing cookies were significantly higher than the other cookies. The cohesiveness was in the range of 0.45–0.60 for all the cookies, however the high gumminess of Asterix starch-based cookies was attributed to high hardness of the Asterix cookies which directly influenced the gumminess. Springiness of all the gluten free cookies was increased after the addition of MS. The hardness is used to characterize the textural properties of baked cookies. Previous studies have reported a decrease in the hardness of cookies after the incorporation of resistant starch [32]. The decrease in the hardness of cookies containing MS was attributed to retarded gelatinization of starch [33].

**Table 4:** Effect of different types of potato starches on the textural properties of gluten free cookies.

Gluten free cookies	Hardness (N)	Cohesiveness	Gumminess (N)	Springiness
Control	194.99 ± 4.98^b^	0.50 ± 0.05^ab^	90.67 ± 6.13^cd^	0.57 ± 0.02^ab^
Native Asterix	218.73 ± 11.58^a^	0.59 ± 0.05^a^	133.82 ± 8.30^a^	0.38 ± 0.05^cd^
Native Kruda	209.83 ± 5.55^a^	0.51 ± 0.06^ab^	116.17 ± 7.89^b^	0.42 ± 0.08^bcd^
Native Mosaic	180.16 ± 4.48^c^	0.46 ± 0.05^b^	86.61 ± 9.99^cd^	0.29 ± 0.04^d^
Modified Asterix	156.69 ± 4.99^d^	0.60 ± 0.09^a^	98.47 ± 3.61^c^	0.64 ± 0.15^a^
Modified Kruda	192.57 ± 6.73^bc^	0.54 ± 0.04^ab^	100.03 ± 8.29^c^	0.52 ± 0.07^abc^
Modified Mosaic	162.78 ± 6.71^d^	0.45 ± 0.04^b^	81.11 ± 7.10^d^	0.46 ± 0.04^bc^

Different small superscripts (a–d) within a column indicate mean values which are significantly different (p < 0.05).

#### 3.2.9 Sensory analysis

Overall, there was no significant difference observed in the sensory attributes of control, native and MS containing cookies (Table 5). However, cookies with modified Asterix starch presented low sensory scores (texture, odor, color, sweetness, crispiness, taste appearance and overall acceptability) in comparison to native Asterix starch cookies. The crispiness score of modified Kruda and modified Mosaic starch containing cookies was lower than respective native starch cookies. However, the overall acceptability scores of gluten-free cookies containing modified Mosaic and Kruda starch were higher than the native starch (Asterix and Mosaic) containing cookies. The addition of resistant starch was reported to have minimal effect on sensory attributes and its addition in low proportion may increase some sensory parameters of food items [34]. These findings support that MS can be used as an alternative to traditional fibers which significantly decrease the sensory acceptance of food products [33].

**Table 5:** Sensory analysis of gluten free cookies.

Sensory attributes	Control	Asterix	Modified Asterix	Kruda	Modified Kruda	Mosaic	Modified Mosaic
Texture	6.12 ± 1.69^a^	6.72 ± 1.1^a^	6.16 ± 1.72^a^	5.84 ± 1.65^a^	6.2 ± 1.58^a^	6.6 ± 1.29^a^	6.56 ± 1.36^a^
Odor	6.76 ± 1.3^a^	6.36 ± 1.44^a^	5.88 ± 1.51^a^	6.16 ± 1.60^a^	6.32 ± 1.34^a^	6.16 ± 1.24^a^	6.36 ± 1.58^a^
Sweetness	6.28 ± 1.67^a^	6.68 ± 1.52^a^	5.92 ± 1.85^a^	5.96 ± 1.74^a^	6.24 ± 1.54^a^	6.44 ± 1.64^a^	6.28 ± 1.59^a^
Crispiness	6.04 ± 2.38^ab^	6.72 ± 1.65^ab^	5.36 ± 1.75^b^	6.68 ± 1.49^ab^	6.4 ± 1.44^ab^	6.96 ± 1.54^a^	6.8 ± 1.58^ab^
Color	7.24 ± 1.53^a^	6.56 ± 1.39^a^	6.44 ± 1.80^a^	6.12 ± 1.96^a^	6.24 ± 1.39^a^	6.68 ± 1.79^a^	6.68 ± 1.74^a^
Appearance	6.56 ± 1.61^a^	6.32 ± 1.52^a^	6.32 ± 1.70^a^	5.68 ± 2.05^a^	6.16 ± 1.55^a^	6.4 ± 1.91^a^	6.4 ± 1.75^a^
Taste	5.84 ± 1.93^a^	6.64 ± 1.58^a^	6.04 ± 1.88^a^	6 ± 1.71^a^	5.76 ± 2.16^a^	6.36 ± 1.95^a^	6.52 ± 2.20^a^
Overall acceptability	6.4 ± 1.66^a^	6.92 ± 1.22^a^	6.32 ± 1.52^a^	6.32 ± 1.62^a^	6.44 ± 1.56^a^	6.64 ± 1.55^a^	6.72 ± 1.54^a^

Different small superscripts within a row indicate mean values which are significantly different (p < 0.05).

## 4 Conclusion

Low-grade potatoes can be used to extract starch and develop a modified starch to use as dietary fiber in food products. The combination of acid hydrolysis and heat treatment significantly increase the amylose content of potato starch. The incorporation of modified starch in food products not only increases the functional value but also results in minimal changes in textural and sensory attributes. Therefore, modified starch can be used as a dietary fiber in food products to replace the conventional fibers, which significantly compromise texture and sensory attributes.

## Ethics statement

Experiments were conducted according to established ethical guidelines, and informed consent obtained from the participants.

## Author contribution statement

Syed Mueez Ali: Performed the experiments; Analyzed and interpreted the data; Contributed reagents, materials, analysis tools or data.

Yumna Siddique: Performed the experiments.

Samina Mehnaz: Contributed reagents, materials, analysis tools or data.

Muhammad Bilal Sadiq: Conceived and designed the experiments; Analyzed and interpreted the data; Contributed reagents, materials, analysis tools or data; Wrote the paper

## Funding statement

This research did not receive any specific grant from funding agencies in the public, commercial, or not-for-profit sectors.

## Data availability statement

Data will be made available on request.

## Declaration of competing interest

The authors declare that they have no known competing financial interests or personal relationships that could have appeared to influence the work reported in this paper.
